# 2,4-Bis(4-eth­oxy­phen­yl)-3-aza­bicyclo­[3.3.1]nonan-9-one

**DOI:** 10.1107/S1600536812037385

**Published:** 2012-09-05

**Authors:** Dong Ho Park, V. Ramkumar, P. Parthiban

**Affiliations:** a Department of Biomedicinal Chemistry, Inje University, Gimhae, Gyeongnam 621 749, Republic of Korea; b Department of Chemistry, IIT Madras, Chennai 600 036, TamilNadu, India

## Abstract

The title compound, C_24_H_29_NO_3_, exists in a twin-chair conformation with an equatorial orientation of the 4-eth­oxy­phenyl groups. The benzene rings are inclined to each other at an angle of 28.0 (1)°. In the crystal, weak C—H⋯O inter­actions link mol­ecules related by translation into chains along the *b* axis. The crystal packing exhibits π–π inter­actions between the benzene rings of neighbouring mol­ecules [centroid–centroid distance = 3.692 (3) Å].

## Related literature

For the synthesis and stereochemistry of 3-aza­bicyclo­[3.3.1]nonan-9-ones, see: Park *et al.* (2011*a* ▶). For the biological activity of 3-aza­bicyclo­[3.3.1]nonan-9-ones, see: Barker *et al.* (2005 ▶); Parthiban *et al.* (2009 ▶, 2010*a* ▶,*b* ▶, 2011*a* ▶). For related structures, see: Parthiban *et al.* (2011*b* ▶); Park *et al.* (2012 ▶).
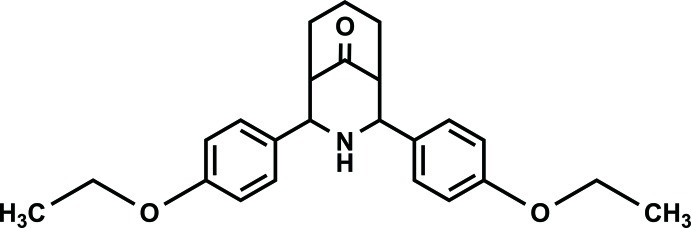


## Experimental

### 

#### Crystal data

C_24_H_29_NO_3_
*M* *_r_* = 379.48
Monoclinic, 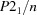

*a* = 14.0319 (11) Å
*b* = 7.3143 (6) Å
*c* = 20.5820 (17) Å
β = 106.841 (3)°
*V* = 2021.8 (3) Å^3^
*Z* = 4
Mo *K*α radiation
μ = 0.08 mm^−1^
*T* = 293 K
0.35 × 0.28 × 0.25 mm

#### Data collection

Bruker APEXII CCD area-detector diffractometer
Absorption correction: multi-scan (*SADABS*; Bruker, 2004 ▶) *T*_min_ = 0.972, *T*_max_ = 0.980
15180 measured reflections
5415 independent reflections
3042 reflections with *I* > 2σ(*I*)
*R*_int_ = 0.034

#### Refinement

*R*[*F* ^2^ > 2σ(*F* ^2^)] = 0.072
*wR*(*F* ^2^) = 0.232
*S* = 1.05
5415 reflections
255 parameters
H-atom parameters constrained
Δρ_max_ = 0.49 e Å^−3^
Δρ_min_ = −0.51 e Å^−3^

### 

Data collection: *APEX2* (Bruker, 2004 ▶); cell refinement: *SAINT* (Bruker, 2004 ▶); data reduction: *SAINT*; program(s) used to solve structure: *SHELXS97* (Sheldrick, 2008 ▶); program(s) used to refine structure: *SHELXL97* (Sheldrick, 2008 ▶); molecular graphics: *ORTEP-3* (Farrugia, 1997 ▶); software used to prepare material for publication: *SHELXL97*.

## Supplementary Material

Crystal structure: contains datablock(s) global, I. DOI: 10.1107/S1600536812037385/cv5330sup1.cif

Structure factors: contains datablock(s) I. DOI: 10.1107/S1600536812037385/cv5330Isup2.hkl

Supplementary material file. DOI: 10.1107/S1600536812037385/cv5330Isup3.cml

Additional supplementary materials:  crystallographic information; 3D view; checkCIF report

## Figures and Tables

**Table 1:** Hydrogen-bond geometry (Å, °)

*D*—H⋯*A*	*D*—H	H⋯*A*	*D*⋯*A*	*D*—H⋯*A*
C18—H18⋯O1^i^	0.93	2.57	3.428 (3)	154
C14—H14⋯O1^i^	0.92	2.61	3.501 (3)	159

Symmetry code: (i) 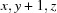.

**Fig. 1.**
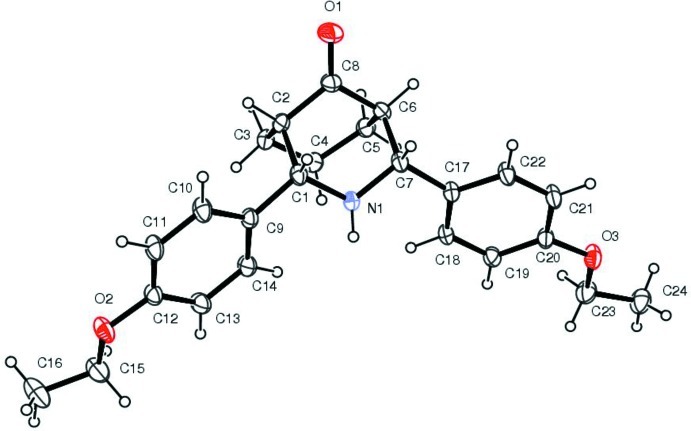
Anistropic displacement representation of the molecule with atoms represented with 30% probability ellipsoids.

## supplementary crystallographic information

### Comment

Alkaloids with 3-azabicyclononane nucleus display broad-spectrum of biological activities ranging from antibacterial to anticancer (Barker *et al.*, 2005; Parthiban *et al.*, 2009, 2010*a*, 2010*b*, 2011*a*). Hence, the synthesis of new molecules that contain 3-azabicyclononane pharmacophore as well as their isolaton from the natural products are important in the field of medicinal chemistry. Accordingly, we synthesized the title compound by a non-laborious method to explore its stereochemistry in the solid-state.

Examination of the asymmery parameters and torsion angles of the title compound reveal that the values are similar to those observed in the analogs *viz*., 2,4-*bis*(4-ethoxyphenyl)-7-methyl-3-azabicyclo[3.3.1]nonan-9-one (Park *et al.*, 2012) and 2,4-*bis*(2-ethoxyphenyl)-7-methyl -3-azabicyclo[3.3.1]nonan-9-one (Parthiban *et al.*, 2011*b*). The torsion angles of the title compound C2—C8—C6—C7, C1—C2—C8—C6, C2—C8—C6—C5 and C3—C2—C8—C6 are -62.5 (2), 62.3 (2), 62.6 (2) and -62.6 (2)°, respectively, that clearly assign the chair-chair conformation to the bicycle as in the analogs. The orientations of the ethoxyphenyl groups on both sides of the secondary amino group are identified by their torsion angles. The torsion angles C8—C2—C1—C9 and C8—C6—C7—C17 are 179.34 (18) and -178.52 (18)°, respectively. This clearly conform their equatorial orientations and it is very similar to those in 7-methylated 4-ehtoxyphenyl [C3—C2—C1—C7 and its mirror image is 176.7 (5)%, center of symmetry bisects the molecule] and 2-ethoxyphenyl analogs [C8—C6—C7—C15 and C8—C2—C1—C9 are 176.83 (14) and -179.07 (14)°, respectively]. In the title compound, two benzene rings are inclined to each other with an angle of 28.0 (1)° as in 7-methylated analog (26.11 (3)°), while in 7-methylated *ortho* analog this angle is 12.41 (4)°.

The crystal packing is stabilized by the weak intermolecular C—H···O hydrogen bonds (Table 1) and π–π interactions.

### Experimental

The 2,4-*bis*(4-ethoxyphenyl)-3-azabicyclo[3.3.1]nonan-9-one was synthesized by a modified and an optimized Mannich condensation in one-pot, using 4-ethoxybenzaldehyde (0.1 mol, 15.018 g/13.91 ml), cyclohexanone (0.05 mol, 4.90 g/5.18 ml) and ammonium acetate (0.075 mol, 5.78 g) in a 50 ml of absolute ethanol (Park *et al.*, 2011). The mixture was gently warmed on a hot plate at 303–308 K (30–35° C) with moderate stirring till the complete consumption of the starting materials, which was monitored by TLC. At the end, the crude azabicyclic ketone was separated by filtration and gently washed with 1:5 cold ethanol-ether mixture. X-ray diffraction quality crystals of the title compound were obtained by slow evaporation from ethanol.

### Refinement

All hydrogen atoms were fixed geometrically and allowed to ride on the parent carbon atoms with aromatic C—H = 0.93 Å, aliphatic C—H = 0.98 Å, methylene C—H = 0.97 Å, and N—H = 0.86 Å, and with *U*_iso_(H) = 1.2-1.5*U*_eq_(C, N).

### Crystal data

**Table:**

C_24_H_29_NO_3_	*F*(000) = 816
*M**_r_* = 379.48	*D*_x_ = 1.247 Mg m^−^^3^
Monoclinic, *P*2_1_/*n*	Mo *K*α radiation, λ = 0.71073 Å
Hall symbol: -P 2yn	Cell parameters from 4030 reflections
*a* = 14.0319 (11) Å	θ = 2.8–28.3°
*b* = 7.3143 (6) Å	µ = 0.08 mm^−^^1^
*c* = 20.5820 (17) Å	*T* = 293 K
β = 106.841 (3)°	Block, colourless
*V* = 2021.8 (3) Å^3^	0.35 × 0.28 × 0.25 mm
*Z* = 4	

### Data collection

**Table:**

Bruker APEXII CCD area-detector diffractometer	5415 independent reflections
Radiation source: fine-focus sealed tube	3042 reflections with *I* > 2σ(*I*)
Graphite monochromator	*R*_int_ = 0.034
phi and ω scans	θ_max_ = 29.5°, θ_min_ = 2.1°
Absorption correction: multi-scan (*SADABS*; Bruker, 2004)	*h* = −19→10
*T*_min_ = 0.972, *T*_max_ = 0.980	*k* = −6→10
15180 measured reflections	*l* = −26→28

### Refinement

**Table:**

Refinement on *F*^2^	Primary atom site location: structure-invariant direct methods
Least-squares matrix: full	Secondary atom site location: difference Fourier map
*R*[*F*^2^ > 2σ(*F*^2^)] = 0.072	Hydrogen site location: inferred from neighbouring sites
*wR*(*F*^2^) = 0.232	H-atom parameters constrained
*S* = 1.05	*w* = 1/[σ^2^(*F*_o_^2^) + (0.1095*P*)^2^ + 0.8855*P*] where *P* = (*F*_o_^2^ + 2*F*_c_^2^)/3
5415 reflections	(Δ/σ)_max_ = 0.001
255 parameters	Δρ_max_ = 0.49 e Å^−^^3^
0 restraints	Δρ_min_ = −0.51 e Å^−^^3^

### Special details

**Table:**

Geometry. All e.s.d.'s (except the e.s.d. in the dihedral angle between two l.s. planes) are estimated using the full covariance matrix. The cell e.s.d.'s are taken into account individually in the estimation of e.s.d.'s in distances, angles and torsion angles; correlations between e.s.d.'s in cell parameters are only used when they are defined by crystal symmetry. An approximate (isotropic) treatment of cell e.s.d.'s is used for estimating e.s.d.'s involving l.s. planes.
Refinement. Refinement of *F*^2^ against ALL reflections. The weighted *R*-factor *wR* and goodness of fit *S* are based on *F*^2^, conventional *R*-factors *R* are based on *F*, with *F* set to zero for negative *F*^2^. The threshold expression of *F*^2^ > σ(*F*^2^) is used only for calculating *R*-factors(gt) *etc*. and is not relevant to the choice of reflections for refinement. *R*-factors based on *F*^2^ are statistically about twice as large as those based on *F*, and *R*- factors based on ALL data will be even larger.

### Fractional atomic coordinates and isotropic or equivalent isotropic displacement parameters (Å^2^)

**Table:**

	*x*	*y*	*z*	*U*_iso_*/*U*_eq_	
O3	1.11887 (13)	0.5928 (3)	0.47084 (8)	0.0489 (5)	
O2	0.82074 (15)	0.5864 (3)	−0.14064 (8)	0.0496 (5)	
O1	0.86995 (18)	−0.2221 (3)	0.17033 (10)	0.0645 (7)	
N1	0.96262 (14)	0.2816 (3)	0.16513 (8)	0.0340 (5)	
H1N	0.9683	0.3986	0.1645	0.041*	
C20	1.08546 (17)	0.5034 (4)	0.40974 (11)	0.0368 (5)	
C5	0.80462 (19)	0.1721 (4)	0.23518 (11)	0.0388 (6)	
H5A	0.7556	0.0889	0.2435	0.047*	
H5B	0.8242	0.2559	0.2733	0.047*	
C18	0.98813 (17)	0.4710 (3)	0.29306 (11)	0.0385 (6)	
H18	0.9428	0.5203	0.2548	0.046*	
C9	0.90291 (17)	0.2906 (3)	0.04047 (10)	0.0326 (5)	
C21	1.12530 (19)	0.3309 (4)	0.40653 (11)	0.0424 (6)	
H21	1.1720	0.2828	0.4444	0.051*	
C8	0.86704 (19)	−0.0568 (3)	0.16953 (12)	0.0385 (6)	
C3	0.74291 (18)	0.1672 (4)	0.10705 (11)	0.0377 (5)	
H3A	0.7253	0.2478	0.0679	0.045*	
H3B	0.6878	0.0834	0.1027	0.045*	
C6	0.89630 (19)	0.0616 (3)	0.23207 (11)	0.0370 (5)	
H6	0.9168	−0.0175	0.2721	0.044*	
C7	0.98759 (17)	0.1764 (3)	0.22787 (10)	0.0342 (5)	
H7	1.0412	0.0914	0.2270	0.041*	
C2	0.83556 (18)	0.0568 (3)	0.10609 (11)	0.0354 (5)	
H2	0.8178	−0.0252	0.0668	0.042*	
C19	1.01727 (18)	0.5742 (4)	0.35234 (12)	0.0398 (6)	
H19	0.9911	0.6906	0.3535	0.048*	
C14	0.8740 (2)	0.4705 (4)	0.04078 (11)	0.0427 (6)	
H14	0.8732	0.5238	0.0816	0.051*	
C22	1.09565 (18)	0.2309 (4)	0.34725 (11)	0.0403 (6)	
H22	1.1236	0.1163	0.3456	0.048*	
C11	0.8779 (2)	0.3173 (4)	−0.08037 (11)	0.0425 (6)	
H11	0.8806	0.2646	−0.1209	0.051*	
C13	0.8459 (2)	0.5745 (4)	−0.01813 (12)	0.0443 (6)	
H13	0.8265	0.6956	−0.0166	0.053*	
C10	0.90468 (19)	0.2167 (4)	−0.02133 (11)	0.0416 (6)	
H10	0.9245	0.0959	−0.0229	0.050*	
C4	0.75624 (17)	0.2812 (3)	0.17080 (11)	0.0360 (5)	
H4A	0.6918	0.3253	0.1724	0.043*	
H4B	0.7973	0.3866	0.1690	0.043*	
C17	1.02478 (16)	0.2975 (3)	0.28970 (11)	0.0332 (5)	
C12	0.84721 (18)	0.4958 (4)	−0.07936 (11)	0.0364 (5)	
C1	0.92723 (17)	0.1728 (3)	0.10336 (10)	0.0337 (5)	
H1	0.9806	0.0885	0.1013	0.040*	
C23	1.0724 (2)	0.7590 (4)	0.47980 (14)	0.0520 (7)	
H23A	1.0010	0.7412	0.4694	0.062*	
H23B	1.0846	0.8516	0.4494	0.062*	
C24	1.1141 (2)	0.8196 (4)	0.55196 (14)	0.0562 (8)	
H24A	1.0961	0.7332	0.5815	0.084*	
H24B	1.0877	0.9377	0.5576	0.084*	
H24C	1.1854	0.8269	0.5630	0.084*	
C15	0.7732 (3)	0.7581 (4)	−0.14446 (14)	0.0554 (8)	
H15A	0.8209	0.8497	−0.1212	0.067*	
H15B	0.7206	0.7515	−0.1226	0.067*	
C16	0.7307 (3)	0.8097 (5)	−0.21709 (15)	0.0712 (10)	
H16A	0.7821	0.8063	−0.2393	0.107*	
H16B	0.7036	0.9309	−0.2200	0.107*	
H16C	0.6790	0.7251	−0.2388	0.107*	

### Atomic displacement parameters (Å^2^)

**Table:**

	*U*^11^	*U*^22^	*U*^33^	*U*^12^	*U*^13^	*U*^23^
O3	0.0551 (11)	0.0491 (11)	0.0322 (9)	0.0124 (9)	−0.0039 (7)	−0.0091 (8)
O2	0.0672 (12)	0.0548 (12)	0.0289 (9)	0.0118 (10)	0.0172 (8)	0.0084 (8)
O1	0.1000 (18)	0.0292 (11)	0.0569 (13)	0.0043 (11)	0.0110 (11)	0.0016 (9)
N1	0.0411 (10)	0.0356 (11)	0.0225 (9)	−0.0035 (9)	0.0048 (7)	0.0003 (8)
C20	0.0364 (11)	0.0413 (14)	0.0279 (11)	0.0011 (10)	0.0016 (9)	−0.0016 (10)
C5	0.0483 (13)	0.0402 (14)	0.0304 (11)	−0.0065 (11)	0.0153 (10)	−0.0025 (10)
C18	0.0384 (12)	0.0401 (14)	0.0286 (11)	0.0024 (11)	−0.0035 (9)	0.0057 (10)
C9	0.0352 (11)	0.0384 (13)	0.0241 (10)	−0.0015 (10)	0.0085 (8)	−0.0017 (9)
C21	0.0436 (13)	0.0488 (16)	0.0267 (11)	0.0115 (12)	−0.0027 (9)	0.0024 (10)
C8	0.0485 (13)	0.0281 (13)	0.0377 (12)	0.0037 (11)	0.0105 (10)	0.0002 (10)
C3	0.0375 (11)	0.0411 (14)	0.0305 (11)	−0.0038 (11)	0.0034 (9)	−0.0014 (10)
C6	0.0495 (13)	0.0306 (12)	0.0278 (11)	0.0006 (11)	0.0065 (9)	0.0054 (9)
C7	0.0375 (11)	0.0366 (13)	0.0257 (10)	0.0074 (10)	0.0046 (8)	0.0019 (9)
C2	0.0476 (13)	0.0306 (12)	0.0254 (10)	−0.0010 (10)	0.0066 (9)	−0.0062 (9)
C19	0.0410 (12)	0.0341 (13)	0.0375 (12)	0.0066 (11)	0.0004 (10)	0.0015 (10)
C14	0.0645 (16)	0.0398 (15)	0.0251 (11)	−0.0022 (12)	0.0152 (10)	−0.0057 (10)
C22	0.0450 (13)	0.0411 (14)	0.0292 (11)	0.0153 (11)	0.0017 (10)	0.0009 (10)
C11	0.0538 (14)	0.0512 (16)	0.0235 (11)	0.0093 (13)	0.0128 (10)	−0.0033 (10)
C13	0.0688 (17)	0.0344 (14)	0.0318 (12)	0.0025 (13)	0.0180 (11)	−0.0008 (10)
C10	0.0495 (14)	0.0462 (15)	0.0289 (11)	0.0133 (12)	0.0111 (10)	−0.0028 (10)
C4	0.0357 (11)	0.0381 (14)	0.0348 (12)	0.0018 (10)	0.0113 (9)	−0.0032 (10)
C17	0.0327 (11)	0.0384 (13)	0.0252 (10)	0.0025 (10)	0.0033 (8)	0.0011 (9)
C12	0.0415 (12)	0.0432 (14)	0.0259 (10)	−0.0013 (11)	0.0118 (9)	0.0016 (10)
C1	0.0381 (11)	0.0377 (13)	0.0248 (10)	0.0048 (10)	0.0083 (8)	−0.0015 (9)
C23	0.0540 (16)	0.0437 (16)	0.0505 (16)	0.0079 (13)	0.0027 (12)	−0.0104 (13)
C24	0.0613 (17)	0.0534 (18)	0.0479 (16)	0.0040 (15)	0.0060 (13)	−0.0154 (14)
C15	0.073 (2)	0.0520 (18)	0.0429 (15)	0.0098 (15)	0.0185 (14)	0.0102 (13)
C16	0.090 (2)	0.076 (2)	0.0498 (18)	0.026 (2)	0.0241 (16)	0.0267 (17)

### Geometric parameters (Å, º)

**Table:**

O3—C20	1.374 (3)	C7—C17	1.514 (3)
O3—C23	1.416 (3)	C7—H7	0.9800
O2—C12	1.377 (3)	C2—C1	1.555 (3)
O2—C15	1.413 (3)	C2—H2	0.9800
O1—C8	1.209 (3)	C19—H19	0.9300
N1—C7	1.456 (3)	C14—C13	1.388 (3)
N1—C1	1.460 (3)	C14—H14	0.9300
N1—H1N	0.8600	C22—C17	1.395 (3)
C20—C19	1.387 (3)	C22—H22	0.9300
C20—C21	1.389 (4)	C11—C10	1.376 (3)
C5—C4	1.527 (3)	C11—C12	1.377 (4)
C5—C6	1.536 (4)	C11—H11	0.9300
C5—H5A	0.9700	C13—C12	1.390 (3)
C5—H5B	0.9700	C13—H13	0.9300
C18—C17	1.379 (3)	C10—H10	0.9300
C18—C19	1.392 (3)	C4—H4A	0.9700
C18—H18	0.9300	C4—H4B	0.9700
C9—C14	1.378 (3)	C1—H1	0.9800
C9—C10	1.389 (3)	C23—C24	1.497 (3)
C9—C1	1.510 (3)	C23—H23A	0.9700
C21—C22	1.379 (3)	C23—H23B	0.9700
C21—H21	0.9300	C24—H24A	0.9600
C8—C2	1.502 (3)	C24—H24B	0.9600
C8—C6	1.507 (3)	C24—H24C	0.9600
C3—C4	1.520 (3)	C15—C16	1.489 (4)
C3—C2	1.536 (3)	C15—H15A	0.9700
C3—H3A	0.9700	C15—H15B	0.9700
C3—H3B	0.9700	C16—H16A	0.9600
C6—C7	1.555 (3)	C16—H16B	0.9600
C6—H6	0.9800	C16—H16C	0.9600
			
C20—O3—C23	118.68 (18)	C13—C14—H14	119.0
C12—O2—C15	118.45 (19)	C21—C22—C17	121.6 (2)
C7—N1—C1	114.74 (19)	C21—C22—H22	119.2
C7—N1—H1N	122.6	C17—C22—H22	119.2
C1—N1—H1N	122.6	C10—C11—C12	120.0 (2)
O3—C20—C19	124.8 (2)	C10—C11—H11	120.0
O3—C20—C21	116.14 (19)	C12—C11—H11	120.0
C19—C20—C21	119.1 (2)	C12—C13—C14	119.3 (2)
C4—C5—C6	113.94 (19)	C12—C13—H13	120.3
C4—C5—H5A	108.8	C14—C13—H13	120.3
C6—C5—H5A	108.8	C11—C10—C9	121.9 (2)
C4—C5—H5B	108.8	C11—C10—H10	119.1
C6—C5—H5B	108.8	C9—C10—H10	119.1
H5A—C5—H5B	107.7	C3—C4—C5	112.0 (2)
C17—C18—C19	121.6 (2)	C3—C4—H4A	109.2
C17—C18—H18	119.2	C5—C4—H4A	109.2
C19—C18—H18	119.2	C3—C4—H4B	109.2
C14—C9—C10	117.4 (2)	C5—C4—H4B	109.2
C14—C9—C1	122.35 (19)	H4A—C4—H4B	107.9
C10—C9—C1	120.2 (2)	C18—C17—C22	117.6 (2)
C22—C21—C20	120.1 (2)	C18—C17—C7	122.54 (19)
C22—C21—H21	120.0	C22—C17—C7	119.8 (2)
C20—C21—H21	120.0	O2—C12—C11	116.4 (2)
O1—C8—C2	124.4 (2)	O2—C12—C13	124.1 (2)
O1—C8—C6	124.3 (2)	C11—C12—C13	119.5 (2)
C2—C8—C6	111.3 (2)	N1—C1—C9	111.8 (2)
C4—C3—C2	114.00 (18)	N1—C1—C2	110.00 (18)
C4—C3—H3A	108.8	C9—C1—C2	111.01 (17)
C2—C3—H3A	108.8	N1—C1—H1	108.0
C4—C3—H3B	108.8	C9—C1—H1	108.0
C2—C3—H3B	108.8	C2—C1—H1	108.0
H3A—C3—H3B	107.6	O3—C23—C24	108.7 (2)
C8—C6—C5	108.31 (19)	O3—C23—H23A	109.9
C8—C6—C7	106.74 (19)	C24—C23—H23A	109.9
C5—C6—C7	115.6 (2)	O3—C23—H23B	109.9
C8—C6—H6	108.7	C24—C23—H23B	109.9
C5—C6—H6	108.7	H23A—C23—H23B	108.3
C7—C6—H6	108.7	C23—C24—H24A	109.5
N1—C7—C17	111.9 (2)	C23—C24—H24B	109.5
N1—C7—C6	110.13 (17)	H24A—C24—H24B	109.5
C17—C7—C6	110.99 (18)	C23—C24—H24C	109.5
N1—C7—H7	107.9	H24A—C24—H24C	109.5
C17—C7—H7	107.9	H24B—C24—H24C	109.5
C6—C7—H7	107.9	O2—C15—C16	109.1 (2)
C8—C2—C3	108.46 (19)	O2—C15—H15A	109.9
C8—C2—C1	107.17 (18)	C16—C15—H15A	109.9
C3—C2—C1	115.2 (2)	O2—C15—H15B	109.9
C8—C2—H2	108.6	C16—C15—H15B	109.9
C3—C2—H2	108.6	H15A—C15—H15B	108.3
C1—C2—H2	108.6	C15—C16—H16A	109.5
C20—C19—C18	119.9 (2)	C15—C16—H16B	109.5
C20—C19—H19	120.0	H16A—C16—H16B	109.5
C18—C19—H19	120.0	C15—C16—H16C	109.5
C9—C14—C13	121.9 (2)	H16A—C16—H16C	109.5
C9—C14—H14	119.0	H16B—C16—H16C	109.5
			
C23—O3—C20—C19	8.5 (4)	C14—C9—C10—C11	−0.6 (4)
C23—O3—C20—C21	−172.5 (3)	C1—C9—C10—C11	176.5 (2)
O3—C20—C21—C22	179.7 (2)	C2—C3—C4—C5	−46.6 (3)
C19—C20—C21—C22	−1.2 (4)	C6—C5—C4—C3	46.6 (3)
O1—C8—C6—C5	−118.4 (3)	C19—C18—C17—C22	−2.5 (4)
C2—C8—C6—C5	62.6 (2)	C19—C18—C17—C7	174.5 (2)
O1—C8—C6—C7	116.5 (3)	C21—C22—C17—C18	2.7 (4)
C2—C8—C6—C7	−62.5 (2)	C21—C22—C17—C7	−174.5 (2)
C4—C5—C6—C8	−53.9 (3)	N1—C7—C17—C18	35.2 (3)
C4—C5—C6—C7	65.7 (3)	C6—C7—C17—C18	−88.3 (3)
C1—N1—C7—C17	179.35 (18)	N1—C7—C17—C22	−147.8 (2)
C1—N1—C7—C6	−56.7 (2)	C6—C7—C17—C22	88.7 (3)
C8—C6—C7—N1	57.0 (2)	C15—O2—C12—C11	169.6 (3)
C5—C6—C7—N1	−63.5 (2)	C15—O2—C12—C13	−11.4 (4)
C8—C6—C7—C17	−178.52 (18)	C10—C11—C12—O2	−179.3 (2)
C5—C6—C7—C17	61.0 (2)	C10—C11—C12—C13	1.6 (4)
O1—C8—C2—C3	118.4 (3)	C14—C13—C12—O2	179.9 (2)
C6—C8—C2—C3	−62.6 (2)	C14—C13—C12—C11	−1.2 (4)
O1—C8—C2—C1	−116.7 (3)	C7—N1—C1—C9	−179.96 (18)
C6—C8—C2—C1	62.3 (2)	C7—N1—C1—C2	56.2 (2)
C4—C3—C2—C8	54.2 (3)	C14—C9—C1—N1	−27.4 (3)
C4—C3—C2—C1	−65.9 (3)	C10—C9—C1—N1	155.7 (2)
O3—C20—C19—C18	−179.6 (2)	C14—C9—C1—C2	95.9 (3)
C21—C20—C19—C18	1.4 (4)	C10—C9—C1—C2	−81.1 (3)
C17—C18—C19—C20	0.5 (4)	C8—C2—C1—N1	−56.4 (2)
C10—C9—C14—C13	1.0 (4)	C3—C2—C1—N1	64.4 (2)
C1—C9—C14—C13	−176.0 (2)	C8—C2—C1—C9	179.34 (18)
C20—C21—C22—C17	−0.8 (4)	C3—C2—C1—C9	−59.9 (2)
C9—C14—C13—C12	−0.2 (4)	C20—O3—C23—C24	174.3 (2)
C12—C11—C10—C9	−0.8 (4)	C12—O2—C15—C16	−166.9 (2)

### Hydrogen-bond geometry (Å, º)

**Table:**

*D*—H···*A*	*D*—H	H···*A*	*D*···*A*	*D*—H···*A*
C18—H18···O1^i^	0.93	2.57	3.428 (3)	154
C14—H14···O1^i^	0.92	2.61	3.501 (3)	159

Symmetry code: (i) *x*, *y*+1, *z*.
